# Characterizing the Multidimensional Relationship Between Spirituality and Obsessive-Compulsive Disorder: Thematic Analysis

**DOI:** 10.2196/81964

**Published:** 2026-02-05

**Authors:** Nora Yanyi Sun, Sofia Eun-Young Guerra, Mahie Mangesh Patil, Sai Supritha Chakravadhanula, Christopher Pittenger, Terence Ching

**Affiliations:** 1 Department of Statistics, Harvard University, 373 Quincy Mail Center, 58 Plympton St, Cambridge, MA, 02138, United States, 1 904-646-8255; 2 Massachusetts Institute of Technology, Cambridge, MA, United States; 3 Orlando Science High School, Orlando, FL, United States; 4 University of Kansas, Lawrence, KS, United States; 5 Department of Psychiatry, Yale School of Medicine, Yale University, New Haven, CT, United States

**Keywords:** spirituality, obsessive-compulsive disorder, OCD, qualitative, religion, coping

## Abstract

To elucidate the complex relationship between spirituality and obsessive-compulsive disorder (OCD), we performed a qualitative analysis of messages (n=225) referencing spiritualities in r/OCD, a public online peer support forum for people with OCD with over 250,000 users; two central themes emerged: (1) influence of spirituality on OCD symptom manifestation and (2) impact of OCD on relationship with spirituality.

## Introduction

The impact of spiritualities, defined as beliefs in transcendent forces, on experiences of obsessive-compulsive disorder (OCD) is nuanced and heterogeneous. Some quantitative research has found mixed or no correlation between spiritualities and OCD outcomes [1], while other research has linked religion to greater severity of OCD symptoms, particularly in the form of scrupulosity [2]. These discrepancies likely reflect the influence of complex underlying mechanisms, which are difficult to capture given the lack of validated quantitative metrics that specifically measure the relationship between spirituality and OCD.

Qualitative self-report from spiritual people with OCD may yield unique insights into this nuanced relationship. However, existing qualitative research remains restricted by small sample sizes (eg, due to OCD stigma) and deductive methodologies that limit analysis to a specific religion. Reddit’s r/OCD forum, with over 250,000 members and 100‐200 new daily posts, offers a large volume of naturally occurring qualitative data [3]. To investigate how people with OCD describe the role of spirituality in their illness narratives, an exploratory study was conducted analyzing posts from r/OCD that referenced faith in any context. This study hypothesizes that most users will report that spiritual beliefs aggravate obsessions related to moral transgressions, while not impacting or potentially alleviating obsessions with other themes—as spiritual explanations may reduce uncertainty—explaining prior mixed results.

## Methods

### Study Methodology

All new threads added to the subreddit within a 4-week period at the start of the study (August-September 2023; n=3543 threads) were downloaded manually. During the analysis protocol described in Table 1, codes on spiritualities and OCD organically emerged. The first and second author collaboratively identified messages that substantively mentioned both OCD and spiritualities (n=225), and the protocol described in Table 1 was repeated with only these messages.

**Table 1.** The table outlines the six-phase process of thematic analysis as applied to posts from an obsessive-compulsive disorder peer support forum. The phases follow Braun and Clarke’s methodology [4,5], including familiarization, code generation, theme development, and writing.

Phase	Steps taken during each phase
1. Familiarization with the dataset	The first and second authors read all eligible posts. Reflective memos were created during reading to note early impressions, possible codes, and interpretive tensions.
2. Coding of the dataset	The first and second authors developed an initial working codebook after a review of the dataset. Then, they independently coded half of the dataset. Discrepancies were resolved through discussion until consensus was reached. Data saturation was achieved after three-quarters of the dataset was coded, with no new concepts emerging from the data.
3. Generation of initial themes	After the dataset was coded, the first and second authors reviewed each other’s work for internal consistency. During this process, the codebook was finalized. Then, codes were reviewed, grouped, and clustered into provisional themes.
4. Revision of themes	Themes were revised, collapsed, or separated until they demonstrated internal coherence and external heterogeneity [4,5]. The remaining themes were cross-checked against the raw data and full dataset to ensure representativeness and sufficiency.
5. Development of subthemes	Codes within each theme were organized into subthemes, following a similar process to what is outlined in phases 3 and 4.
6. Written analysis	This manuscript reports on the meaning of themes and subthemes, including illustrative quotes for each concept. Connections between these results and existing literature and religious coping were identified. All written analysis is contextualized within Braun and Clarke’s guidance to present themes as active constructions of meaning [4,5].

An inductive and reflexive approach to thematic analysis was adapted; codes were generated without preexisting frameworks while acknowledging coder subjectivity. A discrete meaning unit, such as a sentence or short paragraph, was treated as the coding unit; a single post could contain multiple coded instances if multiple distinct ideas were expressed.

Throughout the process, the two coders—who were not spiritual but were raised in Buddhist and Christian contexts, respectively—actively reflected on how personal experiences influenced their interpretation. As Buddhism is a nontheistic Eastern religion that centers self-cultivation, while Christianity is a theistic Western religion that is highly institutionalized, the coders aimed to minimize bias through leveraging the differing perspectives conferred by their respective religious backgrounds.

### Ethical Considerations

This study (protocol ID 2000036062) received a Not Human Subjects Research Determination from the Yale Institutional Review Board.

## Results

Among 3543 threads, 225 messages from 147 unique threads discussed religiosity; 22 codes were used in a total of 302 instances. Two themes emerged, broadly reflecting how OCD affects spiritualities and how spiritualities affect OCD, with the latter representing the majority of instances (Table 2).

**Table 2.** Themes and subthemes resulting from thematic analysis. Counts report the number of coded instances.

Theme and subtheme		Description
Influence of spiritualities on OCD^a^ symptom manifestation (212/302)		
	Spiritual obsessions (143/212)	Users endorsed obsessions related to their faith, endorsing fears like committing blasphemy, going to hell, and being possessed by the devil.
	Religious compulsions (52/212)	Users performed faith-based rituals—most commonly, atonement-based compulsions like praying, confessing, and repenting to higher powers—to cope with obsessions with spiritual or nonspiritual themes.
	Symptom improvements attributed to faith (17/212)	Users reported that their faith helped them overcome OCD symptoms, such as by helping them persist through treatments or resist performing compulsions.
Impact of OCD on relationship with spiritualities (92/302)		
	Loss of faith due to OCD (22/92)	Continued suffering associated with OCD caused users to lose faith in the existence of higher powers (eg, struggles with theodicy) or in religious institutions that failed to support them.
	Persistent faith despite OCD (70/92)	Users described unwavering faith despite OCD, broadly citing divine providence and omniscience; some specifically suggested that higher powers would be understanding of and/or alleviate their OCD.

a OCD: obsessive-compulsive disorder.

## Discussion

Through analyzing a peer support forum, this study identified a bidirectional relationship between spiritualities and OCD. Most messages described how spiritualities influenced OCD. Aligned with initial hypotheses, most messages included reports of faith-themed moral obsessions and compulsions, while some messages reported that faith helped the user overcome OCD, though it was not captured whether these users were also experiencing moral obsessions. Unexpectedly, other messages discussed OCD’s influence on spirituality. These messages primarily emphasized the individual’s persistent faith, though some messages suggested loss of faith due to OCD experiences.

Messages describing spiritual obsessions reflected rigid moral frameworks and thought-action fusion that is commonly observed with scrupulosity [6]. Meanwhile, users who benefited from spirituality reported that faith in higher powers encouraged them to accept uncertainty, a behavior that aligns with clinical advice for OCD. While prior studies suggested that faith may provide comfort and decrease anxiety, the specific mechanism has yet to be investigated among people with OCD [7]. Potential determinants of whether spiritualities are a source of support or fear should be further studied.

Additionally, organized religion was infrequently mentioned, contradicting prior reports that faith communities play significant roles in the mental health benefits associated with spirituality [8]. As this study primarily captures personal rather than social facets of spiritualities, future studies should investigate how peers, communities, and institutions may moderate this relationship. Altogether, qualitative studies in a larger corpus of data from the forum may help clinicians better address each individual’s complex and unique relationship with their faith and potentially modify harmful relationships [9].

This study is limited in its ability to fully engage with literature on spiritualities and OCD due to space constraints. Additionally, Reddit users are predominantly English-speaking, Western, and male [10]; accordingly, this dataset appears to disproportionately represent Judeo-Christian concepts of God. Future studies should determine whether findings accurately capture experiences of those who endorse other religious or spiritual frameworks (eg, Eastern or secular spirituality). As this study is observational, further investigation is necessary to determine whether relationships between spiritualities and OCD may be correlational, even when users report them to be causative. Finally, it is not possible to confirm if users have OCD or honestly represent their experiences; posts may be intentionally or unintentionally tailored toward certain narratives.

Despite limitations, observing how people with OCD choose to report their relationship with spiritualities offers a unique perspective into the true role of spirituality in their lives. Subthemes like “symptom improvements attributed to faith” and “persistent faith despite OCD” may partially result from users’ desire to be viewed as unwaveringly faithful, even if they experience doubts. Through conducting qualitative interviews with forum users, further studies may help elucidate why users conceptualize and/or publicly discuss their faith in a certain manner. These findings may inform the development of more effective therapeutic frameworks that better cohere with how each individual views their relationship with their spirituality—for example, exposure and response prevention therapy that incorporates religiosity exposures should be designed to not minimize one’s commitment to their faith or offend the sanctity of one’s faith.

## Abbreviations

OCD: obsessive-compulsive disorder

## References

[1] Abramowitz JS, Buchholz JL, Abramowitz JS, McKay D, Storch EA (2020). Handbook of Spirituality, Religion, and Mental Health.

[2] Yorulmaz O, Gençöz T, Woody S (2009). OCD cognitions and symptoms in different religious contexts. J Anxiety Disord.

[3] Sun NY, Pittenger C, Ching T (2025). Analyzing the contents of a large, public online peer support forum for obsessive-compulsive disorder: thematic analysis. JMIR Form Res.

[4] Clarke V, Braun V (2017). Thematic analysis. J Posit Psychol.

[5] Braun V, Clarke V (2019). Reflecting on reflexive thematic analysis. Qualitative Research in Sport, Exercise and Health.

[6] Henderson LC, Stewart KE, Koerner N, Rowa K, McCabe RE, Antony MM (2022). Religiosity, spirituality, and obsessive-compulsive disorder-related symptoms in clinical and nonclinical samples. Psycholog Relig Spiritual.

[7] de Abreu Costa M, Moreira-Almeida A (2022). Religion-adapted cognitive behavioral therapy: a review and description of techniques. J Relig Health.

[8] Perez LG, Cardenas C, Blagg T, Wong EC (2025). Partnerships between faith communities and the mental health sector: a scoping review. PS (Wash DC).

[9] Rosmarin DH Spirituality, Religion, and Cognitive-Behavioral Therapy: A Guide for Clinicians.

[10] Barthel M, Stocking G, Holcomb J, Mitchell A (2016). Reddit news users more likely to be male, young and digital in their news preferences. Pew Research Center.

